# LncRNA SNHG3: a potential biomarker for human diseases

**DOI:** 10.3389/fcell.2026.1720402

**Published:** 2026-01-27

**Authors:** Fu-Jia Ren, Xiao-Yu Cai, Yao Yao, Guo-Ying Fang

**Affiliations:** 1 Department of Pharmacy, Hangzhou Women’s Hospital, Hangzhou, China; 2 Department of Pharmacy, Hangzhou First People’s Hospital, Hangzhou, China; 3 Department of Pharmacy, Women’s Hospital School of Medicine, Zhejiang University, Hangzhou, China

**Keywords:** alternative splicing, biomarker, human diseases, RNA metabolism, SNHG3

## Abstract

With advancements in high-throughput sequencing and molecular biology technologies, the emerging significance of long non-coding RNAs (lncRNAs) in pathological conditions has been progressively unveiled. SNHG3, a member of the small nuclear RNA host gene (SNHG) family, is localized in both the nucleus and cytoplasm, and plays a pivotal role in multiple aspects of RNA metabolism, including transcription, splicing, translation and stability. Accumulating evidence indicates that SNHG3 is implicated in various human diseases, with a predominant focus on its oncogenic functions in different malignancies. Mechanistically, SNHG3 exerts its pathological functions by acting as a miRNA sponge, co-transcription factor or repressor, and stabilizer for oncogenic transcripts. Recent studies have further uncovered the essential role of SNHG3 in neurological disorders, such as brain injury, spinal cord injury, and drug-induced nerve injury. In this review, we comprehensively summarize the involvement of SNHG3 in various human diseases, and highlight its dual potential as a diagnostic and prognostic biomarker. Furthermore, we elucidate the regulatory mechanisms by which SNHG3 influences multiple RNA metabolism processes in related pathological processes, and propose its potential role in alternative splicing and the formation of cytoplasmic or nucleic ribonucleoprotein (RNP) granules.

## 1 Introduction

In recent years, research on the pathogenesis of human diseases has predominantly focused on protein-coding genes (Brandes et al., 2019; Lawrence et al., 2014). However, it is noteworthy that protein-coding sequences constitute less than 2% of the human genome, whereas a large fraction is transcribed into RNA with limited or no protein-coding potential (The ENCODE Project Consortium, 2012). Based on their length, non-coding RNAs (ncRNAs) are broadly classified into two categories: small ncRNAs (<200 nt) and the long ncRNAs (lncRNAs, >200 nt) (Beermann et al., 2016). A substantial repertoire of lncRNAs is transcribed by RNA polymerase II and share structural features with mRNAs, including 5’-capping, splicing, and 3’-polyadenylation (Cabili et al., 2011; McDonel and Guttman, 2019). Despite their lower abundance, limited evolutionary conservation, and lack of protein-coding capacity, lncRNAs play diverse and critical roles in mammalian biology from embryonic development to immune regulation (Yao et al., 2019). Within the nucleus, they often regulate chromatin architecture and remodeling by recruiting transcription factors or modulating nuclear body formation. In the cytoplasm, they regulate mRNA stability, translation, and protein modification by competing with the endogenous RNAs or interacting with protein complexes (Rashid et al., 2016; Yao et al., 2019). Notably, a burgeoning body of experimental evidence highlights the pivotal role of lncRNAs in RNA metabolism and their dysregulation in diverse human diseases, particularly cancers, where they can function as oncogenes or tumor suppressors (Cui et al., 2015a; Cui et al., 2015b; Xin et al., 2018). Given their extensive regulatory functions, systematically cataloging lncRNAs is essential for elucidating their complex roles in pathogenesis.

**FIGURE 1:**
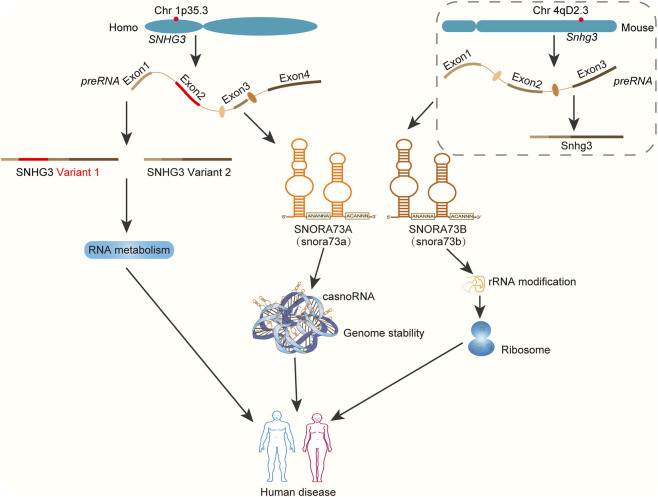
Overview of human *SNHG3*. Human *SNHG3* is located on the human chromosome 1p35.3 and encodes two lncRNA transcripts, namely lncRNA SNHG3 variant 1 (NR_036473.1, 2,346 nt) with four exons and variant 2 (NR_002909.2, 2,238 nt) with three exons. Variant 1 contains an additional exon of 108 bp compared to variant 2. The genomic locus of SNHG3 was present in the common ancestor of human and mouse. SNHG3 plays a crucial role in multiple human diseases through regulating RNA metabolism, such as RNA transcription, stability or translation. SNORA73A/B, encoded within *SNHG3* gene locus, canonically function as modifiers for rRNA, which is essential for the production of efficient and accurate ribosomes. In addition, SNORA73A/B have been recently identified as chromatin-associated orphan snoRNAs (casnoRNAs) that forms a snoRNP with PARP1 and canonical H/ACA proteins DKC1/NHP2, which maintains cancer genome stability. Mouse *Snhg3* is derived from Chromosome 4q2D3, consists of three exons, which is orthologous to human SNHG3 variant 2. This figure is a schematic diagram, which is based on data from relevant references.

Recent years have witnessed growing experimental interest in the small nucleolar RNA host gene (*SNHG*) due to its implications in human diseases (Zimta et al., 2020). The *SNHG3* gene is located on human chromosome 1p35.3 and encodes two distinct lncRNA transcripts. These are lncRNA SNHG3 variant 1 (NR_036473.1, 2,346 nt) with four exons and variant 2 (NR_002909.2, 2,238 nt) with three exons (https://www.ncbi.nlm.nih.gov). Given their distinct structures, it is plausible that these variants exhibit unique, and potentially opposing functions, although current studies have not made a clear function distinction between them. For example, a recent study revealed that A-to-I edited SNHG3 variant 2 promotes non-small cell lung cancer (NSCLC) metastasis by enhancing fatty acid oxidation and resisting ferroptosis (Chen et al., 2025). Importantly, increasing studies reported that SNHG3 plays a pivotal role in embryonic development (Lu et al., 2019), inflammation (Liang et al., 2023; Sun et al., 2021; Wang T. et al., 2022), metabolism (Xie et al., 2024a), cell proliferation and differentiation (Chen et al., 2022; Sun et al., 2022; Tan and Zheng, 2022). Dysregulated levels of SNHG3 contribute to the emergence of various human diseases, particularly tumorigenesis. SNHG3 drives tumor progression in multiple malignancies (e.g., lung, liver and gastric cancers) by promoting tumor cell proliferation, metastasis, chemoresistance, metabolic reprogramming, and tumor microenvironment (TME) remodeling (Xu et al., 2020). Mechanistically, SNHG3 orchestrates pathological processes through regulating RNA metabolism, such as acting as a miRNA sponge (Guo et al., 2024), stabilizing oncogenic transcripts (Huang et al., 2024), and functioning as a co-transcriptional activator or repressor (Chen et al., 2022; Xie et al., 2024b). Notably, an increasing number of studies indicate that SNORA73A and SNORA73B, which are also transcribed from the *SNHG3* gene, play non-redundant roles in lipid metabolism in metabolic dysfunction-associated fatty liver disease (MASLD) (Sletten et al., 2021) and cell differentiation in acute myeloid leukemia (AML) (Han et al., 2022). These studies highlight the multifaceted role of the *SNHG3* gene locus in different pathological processes (Figure 1). Nevertheless, a systematic understanding of lncRNA SNHG3 in disease-associated RNA metabolism remains incomplete, necessitating further investigation.

**FIGURE 2:**
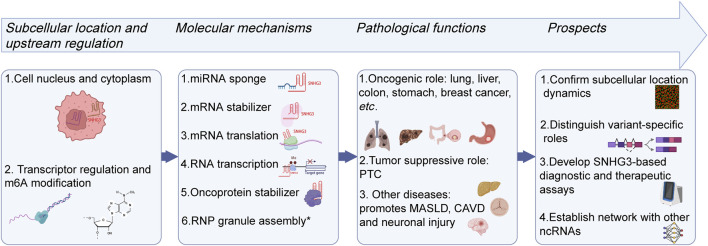
Conceptual framework of SNHG3. This schematic diagram delineates the multifaceted roles of SNHG3 in human diseases, beginning with its subcellular localization and upstream regulation. It further summarizes its molecular mechanisms, with a focus on RNA metabolism, and elucidates its functions across various cancers and other diseases in different organ systems. Finally, the framework highlights future research directions. The asterisk (*) indicates a hypothetical mechanism (Illustrations created with Biorender.com).

This review establishes a conceptual framework to systematize the role of SNHG3 in human diseases (Figure 2). We first outline the biogenesis, localization and upstream regulation of SNHG3, then summarize its function in RNA metabolism, and finally elucidate its pathological roles across different human organ systems. Centered on this framework, we aim to detail the regulatory mechanisms of SNHG3 and its diagnostic and prognostic value in specific diseases, with a particular emphasis on its oncogenic activities, therapeutic prospects and current limitations. We further propose its promising roles in alternative splicing (AS) and RNP granule dynamics.

## 2 Basic biology of SNHG3

SNHG3 is a kind of small nucleolar RNAs (snoRNAs). SnoRNAs are a conserved class of 60–300 nucleotides first identified in the late 1980s (Zimta et al., 2020). Primarily localized in the nucleolus, they guide post-transcriptional modification of ribosomal RNA (rRNA), ensuring accurate ribosome assembly (Matera et al., 2007). Beyond their canonical roles, emerging studies implicate snoRNAs in cellular fate determination and tumorigenesis. For instance, C/D box snoRNA U50 has been demonstrated to exert repressive effects on colony formation prostate cancer (PCa) cells, while its mutation abrogates this effect (Dong et al., 2008). Additionally, SNORA42, an H/ACA box snoRNA, drives the progression of NSCLC and correlates with poor survival (Mei et al., 2012). Certain snoRNA genes contain intronic and exonic sequences with limited protein coding capacity. These transcripts are termed as SNHG, such as GAS5 and ZFAS1 (Williams and Farzaneh, 2012; Liu et al., 2015; Askarian-Amiri et al., 2011). Importantly, an increasing body of experimental research has indicated that SNHGs play a crucial role in various human diseases through regulating the vital RNA metabolism processes.

Especially, SNHG3, also known as U17 small nucleolar RNA host (U17HG), is derived from the human Chr 1p35.3 and is located approximately 9 kb upstream of the *RCC1* (Regulator of Chromosome Condensation 1) gene locus. This region can undergo processing to generate two intronic snoRNAs, namely U17a and U17b RNAs, which belongs to the H/ACA box class of snoRNA (Pelczar and Filipowicz, 1998). By analyzing the sequence of human U17HG and its mouse counterpart, researchers found a striking similarity in their exon/intron organizations, despite lacking protein-coding ability. Further investigation into the 5’-terminus of the U17HG gene revealed that transcription initiates with a C residue followed by a polypyrimidine tract, suggesting that SNHG3 belongs to the 5’-terminal oligopyrimidine family (Pelczar and Filipowicz, 1998). Cell fractionation experiments have identified that SNHG3 is abundant in the cytoplasm of Hela cells (Pelczar and Filipowicz, 1998). However, increasing studies have identified that SNHG3 is distributed across both the cell nucleus and cytoplasm depending on different cell types. Furthermore, the expression of SNHG3 is regulated by multiple factors, including specific transcription factors (e.g., E2F1) and epigenetic mechanisms such as *N6*-methyladenosine (m6A) modification (Ji et al., 2023; Shi et al., 2020). Importantly, SNHG3 exerts broad regulatory control over various RNA metabolism processes, encompassing RNA transcription, stability and translation. With advancements in sequencing and RNA detection technology and experimental strategies, the expression pattern, important function and mechanism of SNHG3 in other human diseases except for cancers has been gradually revealed. This will be addressed in the following section.

## 3 Role of SNHG3 in different RNA metabolism process

### 3.1 Experimentally validated mechanisms of SNHG3

**FIGURE 3:**
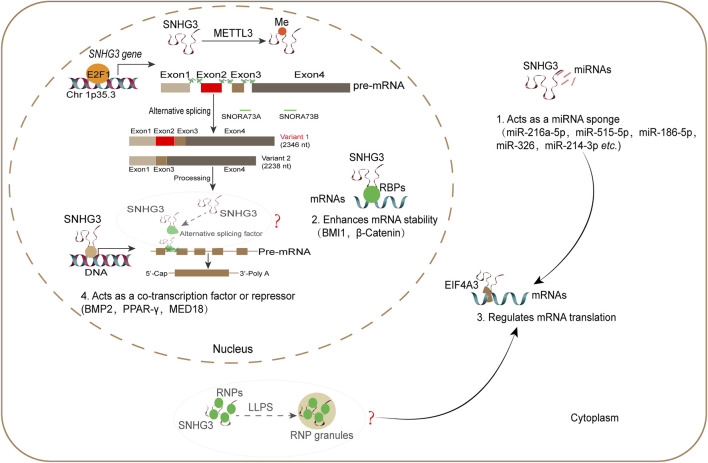
SNHG3 is implicated in different RNA metabolic processes. SNHG3 is broadly localized in the cell nucleus and cytoplasm. In nucleus, SNHG3 acts as a co-transcription factor or repressor, and oncogenic mRNA stabilizer. Alternative splicing regulates the abundance of two SNHG3 variants, SNORA73A and SNORA73B, but which splicing factor is involved in this process is unknown. Besides, SNHG3 is predicted to have multiple binding sites for splicing factors, such as SRSF1 and SRSF2, whether SNHG3 participates in alternative splicing of mRNA is also unknown (hypothesis, represents by question marks). In cytoplasm, SNHG3 has been reported to function as a miRNA sponge or direct mRNA translation regulator through binding EIF4A3, thereby regulating mRNA translation. Furthermore, SNHG3 is predicted to own potential binding sites for RNP granule proteins such as G3BP1 and YTHDF2, it is interesting to investigate the involvement of SNHG3 for RNP granule formation, since P processing bodies and SGs is intimately involved in RNA stability or storage (hypothesis, represents by question marks). This figure is a schematic diagram.

LncRNAs represent a class of regulators involved in various aspects of RNA metabolism process, spanning across nucleus to cytoplasm, encompassing RNA transcription, splicing, stability, translocation, degradation and translation (Bridges et al., 2021; Chang et al., 2022). Recent studies have elucidated the involvement of SNHG3 in different RNA metabolism process (Figure 3). (1) Acts as a miRNA sponge. For example, direct experimental evidence indicates that SNHG3 sequesters critical miRNAs implicated in different cancers, including lung cancer (miR-216a-5p and miR-515-5p), GC (miR-186-5p), HCC (miR-326 and miR-214-3p) (Xu et al., 2020). Thus, SNHG3 indirectly facilitates the expression of downstream mRNAs through regulating miRNAs. (2) Regulates mRNA stability. For example, SNHG3 interacts with c-Myc to promote the stability of *B lymphoma Mo-MLV insertion region (BMI1)* mRNA in bladder cancer. This stabilization consequently upregulates BMI1 protein expression (Xie et al., 2023). Besides, nucleic SNHG3 also enhances the mRNA stability of β-Catenin through facilitating the intranuclear transport of hnRNPC (Huang et al., 2024). (3) Regulates mRNA translation. Most studies have suggested that SNHG3 indirectly enhances mRNA translation by competing for target miRNAs. Interestingly, SNHG3 has been identified as a potential regulator for eukaryotic translation initiation factor 4A3 (EIF4A3) in the energy metabolism of ovarian cancer (OC) cells (Li et al., 2018), suggesting that SNHG3 may be directly involved in mRNA translation. (4) Acts as a transcriptional regulator. For example, it activates *bone morphogenetic protein 2 (BMP2)* and *PPAR-γ* gene transcription by inhibiting the H3K27 trimethylation on chromatin (Chen et al., 2022; Xie et al., 2024b). However, SNHG3 suppresses the transcription of mediator complex subunit 18 (MED18) through promoting the methylation levels of its promoter region (Xuan and Wang, 2019). Thus, SNHG3 exhibits dual effects on mRNA transcription. In addition to its involvement in RNA metabolism process, SNHG3 facilitates the stabilization of oncogenic proteins, such as yes1 associated transcriptional regulator (YAP1) (Zhu et al., 2022). For example, nucleic SNHG3 binds to YAP1, suppressing its ubiquitination process and consequently enhancing the stability of YAP1 in cancer cells. This indirectly promotes YAP-mediated gene transcription (Zhu et al., 2022). Collectively, the evidence discussed above establishes the role of SNHG3 in regulating different RNA metabolism process, and also points to its emerging role in the direct regulation of oncogenic proteins.

### 3.2 An emerging mechanistic hypothesis: SNHG3 as a liquid-liquid phase separation (LLPS) modulator

LLPS is a fundamental mechanism driving the formation of biomolecular condensates such as stress granules (SGs) and nuclear speckles (NSs) involved in RNA metabolism, with broad implications in both physiological and pathological processes (Wang et al., 2021). Notably, a growing body of evidence indicates that lncRNAs (e.g., NEAT1, MEG3 and Xist) can nucleate or modulate LLPS by interacting with proteins harboring intrinsically disordered regions (Ding et al., 2025; Wang et al., 2020; Yao et al., 2025). This prompts an investigation into whether SNHG3 functions similarly. Notably, SNHG3 is predicted to bind multiple RNP granule proteins (https://rnasysu.com/encori/), including the SG components G3BP1 and YTHDF2, as well as the splicing factors SRSF1 and SRSF2 which are known to undergo LLPS during NS formation (Fu and Zhuang, 2020; Li et al., 2023). Therefore, we propose two potential mechanistic hypotheses: SNHG3 could potentially promote SG assembly by facilitating the LLPS of specific proteins, thereby regulating RNA translation. In addition, it might participate in NS dynamics to regulate alternative splicing (Figure 3). Investigating these mechanistic hypotheses could uncover novel aspects of SNHG3’s functionality in RNA metabolism and disease pathogenesis.

## 4 Role of SNHG3 in human diseases

In the past two decades, SNHG3 has been established as a key player in a wide spectrum of human diseases. Its dysregulation is implicated not only in diverse malignancies (Xu et al., 2020) but also in non-cancerous conditions such as neurological disorders (Liang et al., 2023), cardiovascular diseases (Chen et al., 2022), and metabolic diseases (Xie et al., 2024b). Mechanistically, SNHG3 serves as a critical regulator controlling multiple aspects of cellular processes including differentiation, proliferation, apoptosis, immune response and energy metabolism through regulating different RNA metabolism process (Tables 1, 2). In the following section, we will comprehensively address the involvement of SNHG3 in RNA metabolism process associated with specific human diseases across eight physiological systems.

**TABLE 1:** The role and regulatory mechanism of SNHG3 in different human cancers.

Cancer type	Expression	Role	Mechanism	References
NSCLC	Up	Promotes cell proliferation and metastasis	Regulates TGF-β and IL-6/JAK2/STAT3 pathway Acts as a miRNA sponge for miR-216a-5p and miR-515-5p	Shi et al. (2020), Zhao et al. (2020), Li Y. et al. (2021)
GC	Up	Promotes cell proliferation and metastasis	Inhibits transcription of MED18 by facilitating methylation levels Acts as a miRNA sponge for miR-186-5p	Xuan and Wang (2019), Ji et al. (2023)
CRC	Up	Promotes cell proliferation	Regulates hnRNPC-mediated RNA stability of β-catenin Acts as a miRNA sponge for miR-182-5p and miR-34b-5p	Huang et al. (2024), Huang et al. (2017), Zhao et al. (2022)
HCC	Up	Promotes cell proliferation, metastasis, drug resistance and recurrence	Acts as a miRNA sponge for miR-502-3p, miR-128, miR-326, miR-139-5p and miR-214-3p Activates NEIL3 transcription through recruiting E2F1	Guo et al. (2024), Zhang P. F. et al. (2019), Zhao et al. (2019), Wu et al. (2019), Zhan et al. (2021), Zhang et al. (2023)
OC	Up	Promotes cell proliferation and metastasis	Acts as a miRNA sponge for miR-339-5p and miR-139-5p	Liu et al. (2020), Zhang et al. (2021)
BC	Up	Promotes cell proliferation, metastasis, EMT, energy metabolism and osteolysis	Acts as a miRNA sponge for miR-384, miR-154-3p, miR-186-5p, miR-330-5p and miR-1273g-3p	Ma et al. (2020), Jiang et al. (2020), Wan et al. (2021), Li Y. et al. (2020), Sun et al. (2022)
PCa	Up	Promotes cell proliferation and metastasis	Acts as a miRNA sponge for miR-152-3p, miR-577, miR-1827 and miR-487a-3p	Wang X. et al. (2022), Li T. et al. (2020), Hu et al. (2022), Yu and Ren, 2022
ccRCC	Up	Promotes proliferation, metastasis and angiogenesis	Acts as a miRNA sponge for miR-139-5p and miR-10b-5p	Zhang C. et al. (2019), Xu et al. (2021)
Bladder cancer	Up	Promotes cell proliferation, metastasis and EMT	Acts as a miRNA sponge for miR-515-5p Facilitates the stability of BMI1 by binding C-Myc	Dai et al. (2020), Xie et al. (2023)
AML	Up	Promotes cell proliferation	Acts as a miRNA sponge for miR-758-3p	Peng et al. (2020)
PTC	Down	Suppresses cell proliferation and metastasis	Activating AKT/mTOR/ERK pathway	Duan et al. (2020)
Glioma	Up	Promotes cell proliferation and metastasis	Acts as a miRNA sponge for miR-384 and miR-485-5p	Zhang et al. (2020), Guo et al. (2021)
Osteosarcoma	Up	Promotes cell proliferation and metastasis	Acting as a sponge for miRNA-196a-5p and miRNA-151a-3p	Chen et al. (2019), Zheng et al. (2019)

**TABLE 2:** The role and regulatory mechanism of SNHG3 in other human diseases.

Disease type	Expression	Role	Mechanism	References
MASLD	Down	Aggravates hepatic steatosis	SNHG3 reduces H3K27me3 enrichment at the Pparg promoter, thereby enhancing PPARγ expression	Xie et al. (2024b)
CAVD	Up	Promotes osteoblast differentiation of hVICs	Interacts with EZH2 to suppress trimethylation of BMP2 promoter, enhancing BMP2 expression	Chen et al. (2022)
SCI	Up	Promotes cell inflammation and inhibits cell viability	Acts a sponge for miR-139-5p	Wang T. et al. (2022)
Sevoflurane-induced neuronal injury	Up	Promotes neuronal inflammation and pyroptosis	Regulates NEK7 expression by binding to PTPB1	Liang et al. (2023)
Cerebral ischemia-reperfusion injury	Up	Promotes microglial activation and inflammatory factor secretion	Interacts with HADC3 to enhance its stability	Huang D. et al. (2022)
Cerebral ischemic stroke-induced brain injury	Up	Inhibits nerve repair	E2F1 promotes Snhg3 transcription	Sun et al. (2021)

### 4.1 Diseases of respiratory system

In recent years, research on SNHG3 in the respiratory system has primarily focused on lung cancer, which is the leading cause of cancer morbidity and mortality among 36 cancers in 2022 (Bray et al., 2024). The 5-year relative survival rate for lung cancer patients is 27%, with a significant proportion of patients being diagnosed with metastasis due to inadequate screening measures (Siegel et al., 2025). Based on its biological characteristic, lung cancer can be divided into two types, small cell lung cancer and NSCLC. Unfortunately, NSCLC accounts for approximately 85% of all cases, contributing to higher mortality rates (Socinski et al., 2016). Recently, increasing evidence has unveiled the enigmatic role of SNHG in NSCLC (Dong et al., 2015; Lu et al., 2018). Notably, SNHG3 was confirmed to be aberrantly upregulated in NSCLC tissues, which is associated with shorter overall survival (OS). Furthermore, multivariate analysis revealed that SNHG3 is an independent prognostic factor positively correlated with TNM stage (Shi et al., 2020). Despite this significance, the cohort size (*n* = 32) remains limited, necessitating further investigation with a larger patient population to solidify these findings. Functionally, knockdown of SNHG3 in NSCLC cell lines repressed cell proliferation and migration through inhibiting TGF-β pathway and IL-6/JAK2/STAT3 pathway; conversely, overexpression of SNHG3 exerted the opposite effects. Further mechanistic studies indicated E2F1 binds to the promoter region, leading to the transcriptional activation of SNHG3 (Shi et al., 2020). Subsequent studies have demonstrated that SNHG3 functions as a miRNA sponge, and drives NSCLC progression through sequestering miR-216a-5p (Zhao et al., 2020) and miR-515-5p (Li et al., 2021). Consequently, SNHG3 can indirectly promote the expression of key proteins in NSCLC, such as ZEB1 (Zhao et al., 2020) and SUMO2 (Li et al., 2021). Therefore, SNHG3 represents a promising diagnostic biomarker for NSCLC, a finding that requires further validation in larger patient cohorts. However, the precise role of nucleic SNHG3 still remains poorly understood in lung cancer. It is worth investigating whether SNHG3 transcriptionally or post-transcriptionally regulates the expression of crucial genes in the nucleus beyond its role as a miRNA sponge.

### 4.2 Diseases of digestive system

#### 4.2.1 Gastric cancer

**FIGURE 4:**
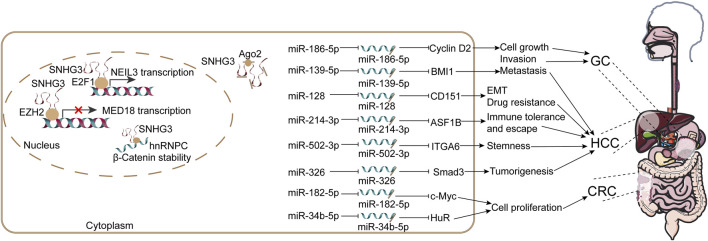
SNHG3 orchestrates RNA metabolism in human digestive system malignancies. On one hand, nucleic SNHG3 regulates RNA transcription by interacting with transcription factors or repressors. SNHG3 recruits EZH2 to the MED18 promoter, thereby facilitating promoter methylation and subsequently inhibiting MED18 transcription and promoting GC progression. Conversely, in HCC, SNHG3 recruits E2F1 to the NEIL3 promoter, promoting NEIL3 transcription. Besides, nucleic SNHG3 promotes the stability of β-Catenin through recruiting hnRNPC. On the other hand, SNHG3 can promote the expression of downstream miRNA genes by binding to miRNA, including miR-186-5p, miR-139-5p, miR-128, miR-214-3p, miR-502-3p, miR-326, miR-182-5p and miR-34b-5p, and then affect cell proliferation, metastasis, EMT, drug resistance and recurrence in gastric cancer, colorectal cancer and HCC. This schematic diagram synthesizes evidence from the published literature.

The role of SNHG3 in digestive diseases has predominantly been investigated in malignant tumors of digestive tract and associated glands (Figure 4). It is widely recognized that GC ranks as the fifth in terms of incidence and mortality (Bray et al., 2024). Due to the generally poor prognosis of GC, early diagnosis is crucial to enable curative treatment (Smyth et al., 2020). Xuan and Wang (2019) reported a significant upregulation of SNHG3 in GC, which was associated with unfavorable prognosis in GC patients, suggesting its potential as a diagnostic biomarker. Functionally, SNHG3 was found to promote GC cell proliferation and metastasis both *in vivo* and *in vitro*. Mechanistic investigations revealed that SNHG3 facilitates the recruitment of EZH2 to the MED18 promoter, elevating its methylation level. This epigenetic silencing represses MED18 transcription and expression, thereby driving the progression of GC (Xuan and Wang, 2019). In addition, SNHG3 has been commonly identified as a miRNA sponge for miR-186-5p, thereby regulating the expression of CyclinD2 in GC (Ji et al., 2023). Collectively, these studies suggest that SNHG3 regulates RNA transcription through acting as a transcriptional repressor or miRNA sponge to control oncogenic transcripts in GC.

#### 4.2.2 Colorectal cancer

The aberrant expression of SNHG3 has been also reported to be indicative of a poor prognosis in patients with colorectal cancer (CRC). Mechanistic studies have revealed that SNHG3 is predominantly localized in the cytoplasm of CRC cells, whereby they promote cell proliferation through sponging miR-182-5p and miR-34b-5p, which leads to the derepression of oncogenic targets like c-Myc and HOXC6, respectively (Huang et al., 2017; Zhao et al., 2022). This activity is further evidenced by a positive correlation between SNHG3 and c-Myc as well as its downstream target genes including CCNB1, CCND2, CDK4 and E2F1 (Huang et al., 2017). Notably, SNHG3 can be shuttled within the tumor microenvironment *via* extracellular vesicles (EVs), either derived from cancer-associated fibroblasts (CAFs) or from CRC cells themselves, thereby amplifying proliferative and metastatic signals, such as enhancing the stability of β-Catenin (Zhao et al., 2022; Huang et al., 2024). Given its detectable presence in patient serum exosomes (Huang et al., 2024), SNHG3 represents a promising non-invasive biomarker for predicting CRC severity.

#### 4.2.3 Liver diseases

Increasing studies indicate that SNHG3 exhibits a functional shift from a metabolic regulator to an oncogenic driver in liver disease progression. While it maintains glucose homeostasis under normal conditions, its expression becomes dysregulated in disease states. In MASLD, SNHG3 is downregulated, yet its overexpression aggravates steatosis *via* modulating PPAR-γ signaling (Xie et al., 2024b). Conversely, SNHG3 is highly upregulated in HCC and serves as an oncogenic driver (Zhang P. F. et al., 2019). This pattern suggests that abnormal SNHG3 expression drives liver pathology, with its function potentially shifting from metabolic regulation to oncogenic promotion during liver disease progression.

Primary liver cancer (PLC) ranks as the sixth most commonly diagnosed cancer and the third leading cause of cancer-related deaths worldwide, imposing significant burdens on human life (Bray et al., 2024). HCC, the most prevalent type of PLC, often arises from risk factors such as chronic hepatitis B virus, hepatitis C virus, drug-induced chronic liver injury, MASLD and alcoholic liver disease (Fujiwara et al., 2018). Despite notable advancements in systemic therapies for HCC, long-term survival rates remain low for patients with severe clinical manifestations due to late-stage diagnosis and a lack of reliable early markers. In recent years, researchers have focused on ncRNAs in HCC diagnosis and prognosis. In particular, many lncRNAs are dysregulated in HCC and, owing to their non-invasive detectability, show great promise as predictive biomarkers (Lim et al., 2019). SNHG3 was reported to be aberrantly upregulated in HCC, which further indicate an unfavorable prognosis among HCC patients (Guo et al., 2024). Besides, SNHG3 was overexpressed in highly metastatic HCC cell lines (HCCLM3), and silence of SNHG3 effectively inhibited HCCLM3 metastasis, as suggested by decreased metastatic cell count and key EMT markers, such as N-cadherin, vimentin and snail. Conversely, overexpression of SNHG3 in a less metastatic cell line PLC/PRF/5 significantly promoted the metastasis ability (Zhang P. F. et al., 2019). Further mechanistic investigations revealed that SNHG3 facilitates stemness of cancer stem cells, EMT and confers sorafenib resistance through acting as a sponge for miR-502-3p and miR-128, thereby leading to dysregulated expression of ITGA6 and CD151 (Guo et al., 2024; Zhang P. F. et al., 2019). Zhao et al. (2019) reported a negative correlation between SNHG3 and miR-326 expression levels in HCC tissues from 47 patients; moreover, the relative expression level of SNHG3 was closely associated with TNM stage. Bioinformatics analysis combined luciferase reporter assay confirmed the combining sites between SNHG3 and miR-326, and mechanistical studies confirmed that the inhibition of miR-326 by SNHG3 aggravated HCC progression both *in vitro* and *in vivo* by positively regulating its target gene SMAD3. Additionally, Wu et al. (2019) demonstrated the oncogenic role of SNHG3 in HCC through silencing experiments conducted in cell lines and nude mice. Interestingly, they found that SNHG3 promotes HCC progression by acting as a miR-139-5p sponge, indirectly upregulating the expression of BMI1, an established oncogene in HCC (Bartucci et al., 2017; Wu et al., 2019). In a separate study, Zhan et al. (2021) constructed a novel lncRNA-miRNA-mRNA network related to the recurrence of HCC and revealed that SNHG3 regulates ASF1B expression by sequestering miR-214-3p, thereby promoting HCC recurrence. Zhang et al. (2023) identified that SNHG3 activates NEIL3 transcription by recruiting E2F1 to the NEIL3 promoter region. Through which, SNHG3 stimulates the proliferation of HCC cells while inhibiting apoptosis and inducing G0/G1 phase to S phase transition.

Collectively, SNHG3 regulates cell proliferation, metastasis, drug resistance and recurrence of HCC through acting as a tumor suppressive miRNA sponge or oncogenic transcription enhancer, and evaluating SNHG3 in HCC patients may present a promising approach to predict tumor progression. However, current evidence is insufficient to establish SNHG3 as a robust prognostic marker in HCC. Although multiple cohorts have consistently demonstrated that SNHG3 is highly expressed in HCC and correlates with adverse clinicopathological features such as advanced tumor stage and poorer overall survival, the modest patient size of these studies limits the strength of this conclusion. Furthermore, a critical unanswered question is whether the two SNHG3 variants differentially regulate miRNAs or exhibit isoform-specific functions during HCC pathogenesis. In addition, the two SNHG3 variants may exhibit distinct subcellular locations. While such localization critically determines whether a lncRNA regulates transcription or translation (Yao et al., 2019), many existing studies have failed to define this for SNHG3. This ambiguity underscores the imperative for future research to precisely ascertain the cellular localization of SNHG3 in HCC cells using advanced techniques, such as single-molecule imaging.

### 4.3 Diseases of reproductive system

**FIGURE 5:**
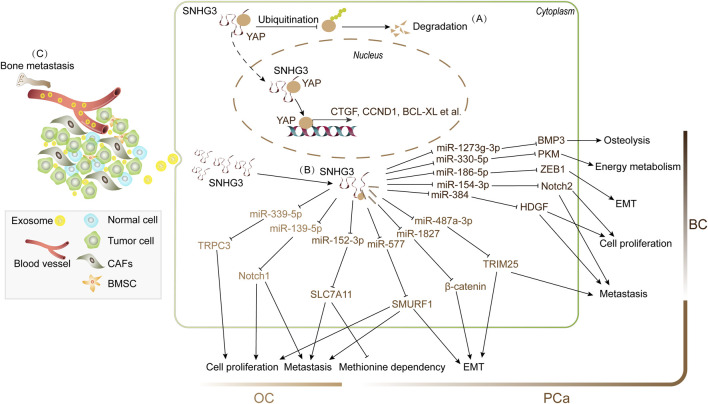
SNHG3 facilitates the progression of human reproductive malignancies by modulating RNA metabolism in key processes including cell proliferation, EMT, methionine dependency and energy metabolism. **(A)** Nucleic SNHG3 indirectly promotes YAP-mediated target gene transcription through impeding YAP ubiquitination and degradation in BC. **(B)** SNHG3 acts as miRNA sponges for miR-339-5p and miR-139-5p in ovarian cancer (OC), miR-152-3p, miR-577, miR-1827 and miR-487a-3p in prostate cancer (PCa), and miR-384, miR-154-3p and miR-186-5p in breast cancer (BC). **(C)** Exosome-derived SNHG3 from cancer-associated fibroblasts (CAFs) promotes energy metabolism of BC through miR-330-5p/PKM axis; differently, exosome-derived SNHG3 from BC cells facilitates bone metastasis of breast cancer and promotes osteolysis of BMSCs through miR-1273g-3p/BMP3 axis. This schematic diagram synthesizes evidence from the published literature.

In recent years, the role of SNHG3 in reproductive system has primarily been investigated in women with cervical cancer (CC) (Zhu et al., 2022), OC (Hong et al., 2018), and BC (Taherian-Esfahani et al., 2019) as well as men with PCa (Li T. et al., 2020). Abnormal upregulation of SNHG3 was observed in these tumor tissues compared to matched normal tissues, and high-risk factors were found to promote its expression. For instance, integration of human papillomaviruses (HPVs) DNA, HPV E6 or E7, resulted in enhanced levels of SNHG3 in Hela and C33A cells, two CC cell lines (Zhu et al., 2022). Furthermore, increased SNHG3 was associated with high malignancy and metastasis rates, as well as low overall survival rates among patients with CC, OC, BC and PCa. In addition to promoting cell proliferation, EMT and metastasis in these malignancies, SNHG3 has been implicated in the regulation of energy metabolism, drug resistance and TME (Li and Zhan, 2020; Li et al., 2018; Li Y. et al., 2020). Mechanistic studies have suggested that SNHG3 plays a regulatory role in the stability of oncoprotein and functions as a sponge for certain tumor suppressive miRNAs. For example, it has been reported that nucleic SNHG3 binds to YAP1, suppressing its ubiquitination process and consequently enhancing the stability of YAP1. This facilitates the binding of YAP1 to its target gene promoters (e.g., CTGF, CCND1, and BCL-XL) in Hela and C33A cells (Zhu et al., 2022). Additionally, most studies have demonstrated that SNHG3 acts as a competitive endogenous RNA by sequestering tumor suppressive miRNAs, such as miR-339-5p (Liu et al., 2020) and miR-139-5p (Zhang et al., 2021) in OC, miR-384 (Ma et al., 2020), miR-154-3p (Jiang et al., 2020) and miR-186-5p (Wan et al., 2021) in BC, and miR-152-3p (Wang X. et al., 2022), miR-577 (Li T. et al., 2020), miR-1827 (Hu et al., 2022) and miR-487a-3p (Yu and Ren, 2022) in PCa, thereby releasing multiple oncogenic transcripts, such as Notch1 (Zhang et al., 2021), and ZEB1 (Wan et al., 2021) (Figure 5). Therefore, SNHG3 exerts its effect on cancer-related genes at both transcriptional and post-transcriptional levels in the development of reproductive system cancers.

SNHG3 was also implicated in the TME in BC. Numerous studies have demonstrated that CAFs within the TME can be recruited and activated by paracrine factors released by tumor cells. Molecular communication between CAFs and cancer cells leads to the release of a plethora of cytokines, thereby regulating tumor growth and metastasis. Li Y. et al. (2020) found that CAFs secrete exosomes enriched with SNHG3, which are internalized by BC cells to reprogram their metabolic pathways. The suppression of this exosomal SNHG3 transfer from CAFs effectively inhibited BC cell proliferation. Mechanistically, SNHG3 functions as a molecular sponge for miR-330-5p, leading the upregulation of pyruvate kinase (PKM) expression. This SNHG3/miR-330-5p/PKM axis consequently suppresses mitochondrial oxidative phosphorylation, enhances glycolytic carboxylation, and ultimately promotes tumor growth. Differently, exosomes derived from BC cells containing abundant SNHG3 were found to enhance bone metastasis in breast cancer (BM-BCa). Knockdown of SNHG3 suppressed the migration of BC cells and inhibited the expression of bone morphogenetic protein 3 (BMP3), an antagonist of other BMPs, in bone marrow mesenchymal stem cells (BMSCs) by upregulating miR-1273g-3p. This subsequently promotes the osteogenic differentiation of BMSCs and bone formation (Sun et al., 2022). Thus, SNHG3 may regulate the microenvironment for tumor bone metastasis through its involvement in intercellular communications in the TME. Further investigations are warranted to elucidate the underlying mechanisms.

### 4.4 Diseases of urinary system

Recently, a bioinformatics study revealed that SNHG3 is upregulated in the peripheral blood in children with Henoch-Schönlein purpura nephritis (HSPN), suggesting its potential role as an immune- and apoptosis- related lncRNA implicated in urinary system disorders (Huang L. et al., 2022). Notably, increasing reports have indicated the regulatory role of SNHG3 in urinary system tumorigenesis. The expression of SNHG3 is aberrantly enriched in clear cell renal cell carcinoma (ccRCC) and bladder cancer. Moreover, elevated levels of SNHG3 are significantly associated with disease progression and unfavorable clinical prognosis in these patients. Functional experiments demonstrated that knockdown of SNHG3 markedly represses cancer cell proliferation, migration, invasion and angiogenesis both *in vitro* and *in vivo*. Mechanistically, in ccRCC, SNHG3 interacts with miR-139-5p and miR-10b-5p to enhance the expression of oncogenes, TOP2A and BIRC5, thereby promoting tumor progression (Xu et al., 2021; Zhang C. et al., 2019). In bladder cancer, SNHG3 positively modulates GINS2 expression by sponging miR-515-5p (Dai et al., 2020). Furthermore, SNHG3 facilitates the stability of BMI1 mRNA by binding to c-Myc or promoting c-Myc expression, ultimately elevating BMI1 protein levels and driving bladder cancer progression (Xie et al., 2023). These studies suggest that SNHG3 functions as an oncogene in ccRCC and bladder cancer through ceRNA mechanism or post-transcriptional stabilization. However, further validation is required to substantiate this hypothesis, necessitating the inclusion of a larger cohort of patient samples.

Despite its potential roles in both inflammatory kidney conditions and urinary cancers, the function of SNHG3 in the inflammation-cancer transition remains largely unexplored. Investigating whether and how SNHG3 mediates the progression from chronic inflammatory states to malignant transformation in the urinary system represents a critical direction for future research.

### 4.5 Diseases of circulatory system

#### 4.5.1 Calcium-soluble aortic valve disease

Calcium-soluble aortic valve disease (CAVD) is the most common heart valve disease, and the differentiation of human valve interstitial cells (hVICs) into osteoblast-like cells plays a pivotal role in CAVD. Recently, aberrant upregulation of SNHG3 has been observed in both human and mouse CAVD, and subsequent *in vitro* and *in vivo* models have confirmed the exacerbating effect of SNHG3 on CAVD progression. Mechanistically, SNHG3 has been identified as an EZH2 decoy that hinders trimethylation of the BMP2 promoter. Consequently, SNHG3 enhances BMP2 expression, thereby activating downstream Smad signaling pathway and promoting osteoblast differentiation of hVICs (Chen et al., 2022). However, it has been reported that under tumorigenic conditions, SNHG3 promotes BMP3 expression in BMSCs, which accelerates osteolysis in BM-BCa (Sun et al., 2022). These findings suggest that SNHG3 can modulate the expression of various BMP family members under different pathological conditions, ultimately influencing TGF-β/Smad signaling and osteogenic differentiation.

#### 4.5.2 Acute myeloid leukemia

SNHG3 is also implicated in hematologic malignancies. Peng et al. (2020) found a significant upregulation of SNHG3 in both AML samples and cell lines, and its high level was correlated with the poor survival of AML patients. Knockdown of SNHG3 inhibited cell proliferation and induced cell apoptosis. Mechanistically, SNHG3 was confirmed to regulate serglycin (SRGN) expression by competitively binding to miR-758-3p, thereby initiating growth promotion functions in AML (Peng et al., 2020). Additionally, transcripts from the *SNHG3* gene locus may have broader regulatory roles: the snoRNA SNORA73, processed from SNHG3, was shown to form non-canonical complexes with DKC1/NHP2 and PARP1, inhibiting PARylation and impairing the DNA damage response in AML cells (Han et al., 2022). These studies suggest that ncRNA originating from the *SNHG3* gene locus has distinct regulatory potential in hematologic malignancies by orchestrating RNA metabolism or DNA stability, potentially through regulating RNP foci formation.

While SNHG3 influences nuclear processes (e.g., epigenetic regulation in CAVD), its cytoplasmic roles (e.g., ceRNA in AML) and the distinct functions of its processed RNAs warrant further investigation across these diseases.

### 4.6 Diseases of the endocrine system

Thyroid cancer is the most prevalent malignancy of the endocrine system, and papillary thyroid carcinoma (PTC) accounts for 90% cases of thyroid cancer (Bray et al., 2024). Intriguingly, in contrast to its well-documented oncogenic roles in other cancers, SNHG3 has been reported to function as a tumor suppressor in PTC (Duan et al., 2020). This study reported that SNHG3 is significantly downregulated in human PTC tissues and cell lines. The expression of SNHG3 was negatively correlated with TNM stage and poor prognosis of PTC patients. Functionally, SNHG3 deficiency promotes the proliferation, migration and invasion of PTC cells *in vitro* and *in vivo* through activating AKT/mTOR/ERK pathway. In a word, SNHG3 can act as a tumor suppressor in the occurrence and development of PTC (Duan et al., 2020), which contradicts its upregulation in many other cancers.

This apparent contradiction presents an intriguing scientific issue. First, the possibility that different transcript variants (V1 vs. V2) of SNHG3, which are not distinguished in most studies, may have opposing functions, and their relative expression might dominate in different cancers. Second, the availability and expression levels of SNHG3’s binding partners (e.g., specific miRNAs or RBPs) can vary dramatically across tissues, leading to fundamentally different functional outcomes. Third, the cellular context is paramount. The signaling environment of PTC, which is frequently driven by mutations in the MAPK pathway, can create a unique landscape where the molecular interactions of SNHG3 lead to tumor-suppressive, rather than oncogenic, outcomes. Importantly, a single study alone cannot definitively establish the tumor suppressive role of SNHG3, and further investigations are warranted to validate this function and to elucidate the regulatory mechanisms by which SNHG3 influences RNA metabolism process in PTC.

### 4.7 Diseases of nervous system

#### 4.7.1 Neurological disorders

Increasing studies have reported that SNHG3 plays a promotive role in brain injury through regulating neuroinflammation. Huang D. et al. (2022) found that SNHG3 is significantly upregulated in the oxygen-glucose deprivation/reoxygenation (OGD/R) cell model and transient middle cerebral artery occlusion (tMCAO) mouse model. Knockdown of SNHG3 ameliorated cerebral ischemia-reperfusion injury through inhibiting microglial activation and secretion of proinflammatory factor, such as IL-6 and TNF-α. Moreover, in cerebral ischemic stroke, the E2F1-mediated upregulation of SNHG3 exacerbates brain injury by suppressing neuronal repair processes (Sun et al., 2021). In addition, serum levels of SNHG3 were significantly upregulated in patients with SCI and showed a negative correlation with miR-139-5p. Mechanistically, SNHG3 functions as a molecular sponge for miR-139-5p, thereby enhancing inflammatory responses and exacerbating neuronal injury (Wang T. et al., 2022). Besides, knockdown of SNHG3 alleviated sevoflurane-induced neuronal injury by restraining the NEK7/NLRP3 axis (Liang et al., 2023). Thus, these findings posit SNHG3 as a likely driver of neuroinflammation and neuronal injury. Nevertheless, research on SNHG3 in neurological disorders remains limited, and there is a lack of large-scale clinical cohorts to validate its immunomodulatory functions.

Noteworthily, emerging studies have begun to explore the diagnostic potential of SNHG3 in neurodegenerative diseases. For example, based on the analysis of the brain region in AD11 transgenic mouse model, SNHG3 has been identified as a potential late-stage specific biomarker for Alzheimer’s disease (AD) (Arisi et al., 2011). Its dysregulation may play a role in AD progression, possibly by regulating gene expression or cellular processes implicated in advanced pathological stages. In multiple sclerosis (MS), clinical data showed that SNHG3 is significantly elevated in patient blood and exhibits high diagnostic potential (AUC = 0.97). Importantly, its expression levels are specifically associated with disease subtypes and age, suggesting a crucial role of SNHG3 in MS pathology (Tayefeh-Gholami et al., 2024). Collectively, these findings highlight SNHG3 as a late-stage marker in AD and a promising diagnostic indicator in MS, reflecting its distinct roles across neurodegenerative and neuroinflammatory conditions. Further functional and mechanistic studies should be performed to exploit its potential for diagnosis, monitoring, or therapy in neurological disorders.

#### 4.7.2 Glioma

SNHG3 is also dysregulated in glioma, with elevated levels in tumor tissues and corresponding decreases in miR-384 and miR-485-5p. Further studies found that SNHG3 promotes the proliferation, migration and invasion of glioma cells through targeting miR-384/HDGF (Zhang et al., 2020) and miR-485-5p/LMX1B axis (Guo et al., 2021). Beyond these cell-autonomous effects, the potential interplay between SNHG3-mediated regulation and the neuroinflammatory tumor microenvironment presents an intriguing avenue for future research into glioma pathogenesis and therapy.

### 4.8 Diseases of motor system

Previously, SNHGs were reported to participate in osteogenic differentiation of BMSCs (Tan and Zheng, 2022). Notably, SNHG3 was identified as a key regulator both in osteolysis (Sun et al., 2022) and osteogenesis (Chen et al., 2022) dependent on disease status, suggesting that ectopic expression of SNHG3 may lead to the occurrence of bone-related diseases. The pathogenesis of osteosarcoma primarily arises from a differentiation defect characterized by the presence of poorly differentiated osteoblasts, predominantly affecting adolescence and childhood (Kansara et al., 2014). Recently, SNHG3 was found to be significantly upregulated in osteosarcoma compared to adjacent normal tissues. Further studies indicated that high expression of SNHG3 predicts a shorter survival of patients with osteosarcoma (Chen et al., 2019; Zheng et al., 2019). Mechanistic studies showed that SNHG3 promotes the expression of HOXC8 through sponging miR-196a-5p, thereby enhancing the growth and metastasis of osteosarcoma cells (Chen et al., 2019). Consistently, Zheng *et al.* found that SNHG3 promotes the expression of RAB22A by absorbing miR-151a-3p (Zheng et al., 2019). These findings suggest that SNHG3 functions as a potential regulator in osteosarcoma through indirectly facilitating oncogenic transcript expression. Given its crucial role in normal bone formation, SNHG3 dysregulation may contribute to the differentiation defect characteristic of osteosarcoma by impeding proper osteoblast maturation. Future investigations should clarify how its genotype influences pathological phenotypes and explore its involvement in other RNA metabolic processes in bone malignancies.

## 5 Discussion

To date, a growing body of evidence has demonstrated that SNHG3 plays an oncogenic role in tumorigenesis through regulating key cellular processes such as proliferation, metastasis and energy metabolism, and aberrant upregulation of SNHG3 predicts poor prognosis of many cancers except for PTC. Nevertheless, the precise molecular mechanisms of SNHG3 are far from understood. For instance, emerging data on the inflammatory regulatory function of SNHG3 suggest its involvement in the TME, and the current study only revealed its role in crosstalk between cancer cells with CAFs and BMSCs. It remains unclear whether SNHG3 modulates the activity of immune cells within the TME, such as macrophages, neutrophils, and T cells, which warrants further investigation.

Beyond its oncogenic roles, increasing evidence suggests that SNHG3 dysregulation is implicated in the pathogenesis of other non-malignant diseases, including neurological disorders, MASLD, and CAVD. This suggests that SNHG3 dysregulation may occur stealthily during early disease stages, disrupting core cellular functions and potentially priming the tissue microenvironment for subsequent malignant transformation. Thus, monitoring SNHG3 expression through applying different detection methods (e.g., RT-qPCR, RNA-seq, *in situ* hybridization) can be a strategy to predict tumorigenesis, and targeting SNHG3, such as developing antisense oligonucleotides (ASOs) or CRISPR-based gene-editing methods, represents a promising approach to delay disease progression. However, the clinical translation of SNHG3-directed therapies faces considerable challenges. Key issues include achieving tissue-specific delivery, minimizing off-target effects of ASOs or gene-editing machinery, improving delivery efficiency, and resolving isoform ambiguity. For example, systemically administered ASOs or gene-editing machinery require sophisticated delivery systems (e.g., ligand-conjugated nanoparticles) to achieve selective accumulation in diseased tissues or cells, thereby minimizing off-target effects and toxicity in healthy organs (Hammond et al., 2021).

Aberrant AS of SNHGs has been demonstrated to induce intron retention, resulting in the downregulation of snoRNAs and exerting an oncogenic role in hepatoblastoma, which is mediated by PABPN1, a splicing factor (Zhen et al., 2023). This discovery implies distinct splicing patterns of SNHGs play a dual role in human pathological processes, with vital splicing factors participating in these processes. Interestingly, SNORA73A and SNORA73B generated by AS of *SNHG3* gene locus were confirmed to promote hepatic steatosis, however the specific splicing factor responsible for regulating the AS of SNHG3, SNORA73A and SNORA73B is not clarified (Sletten et al., 2021). This indicates that the regulatory output of the *SNHG3* gene locus is multi-faceted, extending beyond the production of lncRNA SNHG3. Moreover, the functional implications of two variants of human SNHG3 remain largely unexplored. This is particularly important given emerging evidence that different transcript isoforms from a single lncRNA locus can harbor unique, and even antagonistic functions (Khan et al., 2023). For instance, two splice variants of lncRNA-PXN-AS1 have been shown to exert opposing effects on HCC through regulating PXN mRNA abundance (Yuan et al., 2017). The functional distinction between SNHG3 variants is critical for developing nucleic acid-based therapeutics. To mitigate siRNA off-target risks posed by SNHG3 sequence variations, it is imperative to functionally discriminate between its transcript variants in disease conditions and employ CRISPR-based interference to avoid off-target effects. In addition, specific experimental strategies such as live-cell imaging should be performed to test localization dynamics of SNHG3. Further investigations should be conducted to explore the underlying mechanisms facilitating AS of SNHG3, as well as to elucidate the functional roles of its two transcript variants separately in human diseases.

LncRNAs can be scaffolds for membraneless organelle (MLO) formation, such as paraspeckles, NSs and SGs (Bridges et al., 2021). Given aberrant formation of MLOs in tumorigenesis, it is intriguing whether SNHG3 can serve as a scaffold for MLOs, since SNHG3 possesses multiple binding sites for RNP granule proteins (Tong et al., 2022), and further methods such as CLIP-seq and RIP should be performed to screen and validate potential RNA-binding proteins for SNHG3. As previously reported, SNHG3 is implicated in different RNA metabolism processes such as RNA stability and translation. Notably, SNHG3 exhibits abnormal upregulation in different cancers, while hypoxic environments within tumors induce the formation of SGs, which are the MLOs associated with RNA storage and translation (D’Aiuto et al., 2022). It raises the possibility that SNHG3 has a potential to facilitate the formation of SGs since it has multiple predictive binding sites for G3BP1, a classical marker for SGs (Fu and Zhuang, 2020). Furthermore, whether nucleic SNHG3 is involved in the formation of NSs during RNA splicing since SNHG3 is a possible lncRNA that interacts with AS factor (Figure 3). More interesting aspects regarding RNA metabolism regulatory function of SNHG3 that await further exploration.

Importantly, a comprehensive delineation of the pathogenic function and mechanism of SNHG3, coupled with expanded clinical validation through larger sample sizes, will furnish a more robust theoretical foundation for its establishment as a promising biomarker in human diseases.

## 6 Conclusions and future directions

In conclusion, SNHG3 is widely implicated in various human diseases, with extensive research highlighting its oncogenic functions across multiple malignancies and underscoring its diagnostic and prognostic potential. This review has also provided a comprehensive overview of transcriptional and post-transcriptional functions of SNHG3 in RNA metabolism, including its functions as a miRNA sponge, a co-transcriptional regulator, and a stabilizer of oncogenic transcripts.

While this review consolidates the multifaceted roles of SNHG3 in different cancers and other human diseases, several critical directions for future research should be considered. First, a primary challenge is to move beyond correlative expression studies and confirm the precise subcellular localization dynamics of SNHG3 through experimental strategies. Employing single-molecule imaging techniques across different cell lines will be crucial to understand how its nuclear-cytoplasmic shuttling determines specific functions. Second, the field must distinguish the variant-specific roles of SNHG3. The prevailing assumption that all transcripts function identically is likely inaccurate. Therefore, isoform-specific knockdown/overexpression experiments and the development of variant-sensitive detection methods are imperative. Third, translating this knowledge into clinical impact requires a concerted effort to develop SNHG3-based diagnostic and therapeutic assays. This includes validating SNHG3 as a robust liquid biopsy biomarker and investigating innovative strategies to target its oncogenic interactions, such as developing small molecules, ASOs and CRISPR-based gene-editing. Moreover, employing RBP-focused CLIP-seq experiments to systematically identify shared protein partners between SNHG3 and other RNAs (e.g., circRNAs, piRNAs or snoRNAs) except for miRNAs will be crucial to map the underlying regulatory network and strengthen a systems-level understanding of its function. Addressing these priorities will not only resolve current mechanistic ambiguities but also accelerate the journey of SNHG3 from a compelling molecular player to a validated target and biomarker for human diseases.
